# Mechanisms of Plasticity in Subcortical Visual Areas

**DOI:** 10.3390/cells10113162

**Published:** 2021-11-13

**Authors:** Maël Duménieu, Béatrice Marquèze-Pouey, Michaël Russier, Dominique Debanne

**Affiliations:** INSERM, Aix-Marseille Université, UNIS, 13015 Marseille, France; maeldumenieu@gmail.com (M.D.); beatrice.marqueze@univ-amu.fr (B.M.-P.); michael.russier@univ-amu.fr (M.R.)

**Keywords:** visual system, lateral geniculate nucleus, superior colliculus, synaptic plasticity, intrinsic plasticity, Hebbian plasticity, homeostatic plasticity

## Abstract

Visual plasticity is classically considered to occur essentially in the primary and secondary cortical areas. Subcortical visual areas such as the dorsal lateral geniculate nucleus (dLGN) or the superior colliculus (SC) have long been held as basic structures responsible for a stable and defined function. In this model, the dLGN was considered as a relay of visual information travelling from the retina to cortical areas and the SC as a sensory integrator orienting body movements towards visual targets. However, recent findings suggest that both dLGN and SC neurons express functional plasticity, adding unexplored layers of complexity to their previously attributed functions. The existence of neuronal plasticity at the level of visual subcortical areas redefines our approach of the visual system. The aim of this paper is therefore to review the cellular and molecular mechanisms for activity-dependent plasticity of both synaptic transmission and cellular properties in subcortical visual areas.

## 1. Introduction

### 1.1. Lateral Geniculate Nucleus and Superior Colliculus

**Figure 1:**
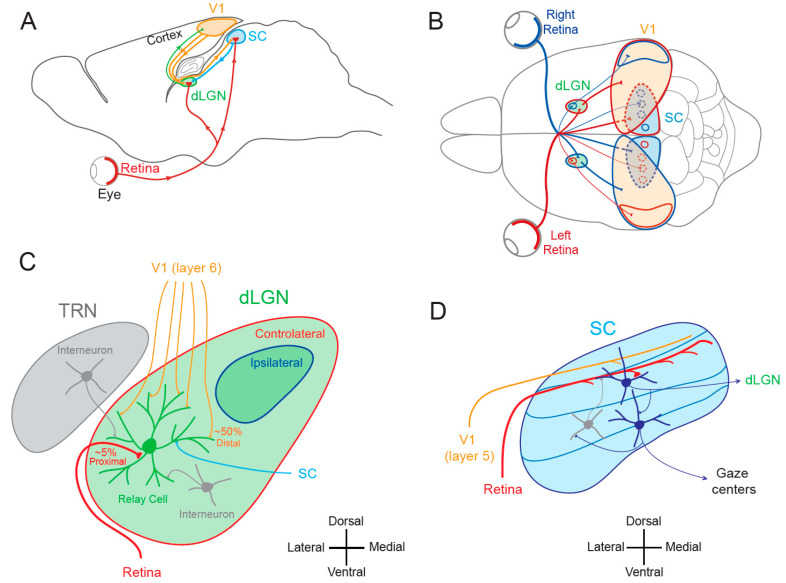
Visual pathways. (**A**) Sagittal view of the rodent visual system. V1, primary visual area; SC, superior colliculus; dLGN, dorsal lateral geniculate nucleus. (**B**) Superior view of the rodent visual system. Red, visual inputs from the left eye. Blue, visual inputs from the right eye. (**C**) Simplified synaptic organization of visual inputs to rodent dLGN. The relay cell receives 3 types of excitatory inputs: (1) small amount (~5%) of functionally powerful contralateral inputs from the retina on proximal dendrite (red), (2) numerous (~50%) but functionally weak feed-back inputs from V1 (orange) on distal dendrites and (3) from the SC (light blue) on medial and distal dendrites. In addition, it is inhibited by interneurons located in the TRN (thalamic reticular nucleus) and in the dLGN (grey). (**D**) Principal inputs and outputs of rodent SC neurons. In the superficial layer, SC neurons receive excitatory inputs from the retina (red) and from V1 (orange) and an inhibitory feed-back (grey) from interneurons located deeper in the SC. Superficial excitatory neurons contact deeper premotor neurons and neurons in the dLGN. Premotor neurons in the deep layer feed gaze centers of the brain. Adapted from [11].

In the mammalian visual system, the dorsal lateral geniculate nucleus (dLGN), a primary recipient structure of retinal inputs at the thalamic level, and the superior colliculus (SC), a lamellar structure involved in the comparison of multi-modal sensory information, constitute the main subcortical visual areas and occupy complementary functions. While the dLGN is involved in precise and conscious vision [1,2,3], the SC is responsible for the initiation of eye and head movements towards specific objects [4,5,6,7]. Both structures receive direct inputs from retinal ganglion cells and from the primary visual cortex and communicate with each other (Figure 1A,B). The dLGN is a thalamic nucleus whose organization varies across species. In primates and cats, the dLGN is organized in alternate monocular layers, whereas in rodents, no such clear lamination is visible. Rather, discrete monocular regions are identified with a large contralateral region surrounding a smaller ipsilateral projection zone (Figure 1C). Two major functional types of dLGN relay neurons are found in primates, cats and rodents: cells that display linear summation (X-type or parvocellular neurons) and cells that display non-linear summation (Y-type or magnocellular neurons) [8,9,10]. The linear type represents the overwhelming majority of dLGN neurons in primates, cats and rodents. 

Receptive fields of visual neurons analyze a portion of visual space and are generally classified according to their response to a positive contrast transition (i.e., ON) or to a negative contrast transition (i.e., OFF). Retinal and thalamic receptive fields are concentric with an ON (or OFF) center and an OFF (or ON) surround. As a consequence, they are weakly or not direction-sensitive. In the primary visual cortex, ON and OFF responses are spatially segregated in simple receptive fields, but they are mixed in complex receptive fields. Cortical receptive fields are usually rectangular and generally direction sensitive.

The dLGN has a retinotopic organization—that is, it follows the mapping of the retina. Remarkably, retinal inputs represent ~5% of the inputs to dLGN relay cells, and cortical inputs from layer 6 neurons represent ~50% of their inputs [2,12]. However, dLGN receptive fields are similar to those of retinal ganglion cells (i.e., monocular ON or OFF center with an OFF or ON surround) and profoundly different from receptive fields of layer 6 cortical neurons (binocular squared and mixed ON-OFF). This surprising mismatch between connection density and functional classification can be explained by several features. First, retinal axons form synapses with relay cells at the proximal dendrites, thus minimizing voltage attenuation along the dendrites whereas cortico-thalamic synapses are located in distal dendrites [2]. In addition, retinal inputs produce a post-synaptic current ~10 times larger than cortical inputs, and retino-geniculate synapses display a higher release probability [2]. Thus, retino-geniculate inputs are classically considered as *driver* inputs, whereas cortico-geniculate inputs are considered as *modulator* inputs [13]. The other inputs to dLGN relay neurons arise from the thalamic reticular nucleus (TRN), from dLGN interneurons and from the SC through the stellate cells [14]. TRN and dLGN interneuron inputs are inhibitory, whereas stellate-SC inputs are excitatory (Figure 1C).

The SC is a three-dimensional structure with sensory inputs organized into a series of laminae that are topographically mapped and aligned with respect to the visual field [4,7]. The superficial layers receive visual inputs, whereas deeper layers receive other sensory and motor inputs [15]. As the dLGN, rodent SC also receives strong inputs from the retina [16], among which about 80% are common to the dLGN [17]. Both dLGN and SC receive different classes of ganglion cells (ON, OFF), but transient responding ganglion cells are more commonly represented in the SC than in the dLGN. In addition, the proteins transported by retinal ganglion cell axons are mostly different in each target [18]. Retina-recipient neurons project onto premotor neurons that activate gaze centers and are inhibited by local interneurons [11,19] (Figure 1D). The activation of premotor neurons located in the deep layers of the SC can trigger saccades [20]. Saccades correspond to gaze shifts that aim to maintain the fovea on the target of interest [21]. Saccades are generally so brief that visual feedback cannot guide them to their targets and thus, the saccadic motor command must be accurately specified upstream the movement.

### 1.2. Cortical and Subcortical Plasticity

Activity-dependent plasticity in the visual system was classically thought to be exclusively expressed at the cortical level [22], whereas subcortical areas such as the dLGN and the SC were traditionally considered to be involved in transmission of visual signals and thus expressing much less to no plasticity. Supporting this idea, monocular deprivation has been thought for a long time to produce no change in receptive field properties of dLGN neurons, dating back to the pioneering work by Wiesel and Hubel in 1963 [23,24,25,26]. Thus, for many years, the dogma was that functional plasticity occurs only in the superior visual areas located in the cerebral cortex where visual information is processed and possibly stored, whereas subcortical areas are only devoted to a rigid processing of visual information. However, this dichotomous view between noble and subaltern visual areas has been challenged by later works indicating that subcortical areas do express functional plasticity and actively participate in both the elaboration of perceptual decision-making and cognitive functions [3,27]. 

## 2. Functional Plasticity in Subcortical Visual Areas

### 2.1. Functional Plasticity in the dLGN

**Figure 2:**
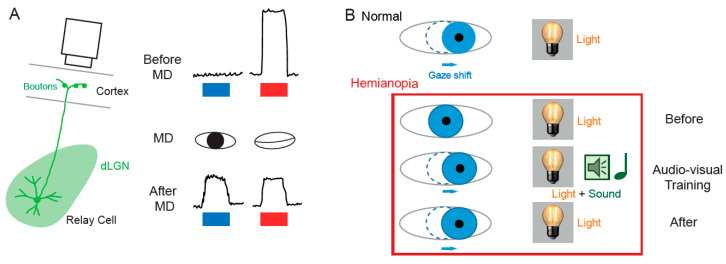
Functional plasticity in subcortical visual areas. (**A**) In the dLGN. Left, calcium imaging setup in presynaptic boutons of dLGN relay cell. Right, calcium signals evoked by visual stimulation (blue bar, stimulation of the right eye; red bar, stimulation of the left eye). Upper part, before MD; middle part, MD; lower part, after MD. Visual response is evoked only through one eye before MD, whereas visual response is evoked through each eye after MD on the left eye (adapted from [38]). (**B**) In the SC. In normal patients, gaze orientation occurs upon visual stimulation (light). In hemianopia patients, the same light stimulus produces no gaze shift. During audio-visual training where light is associated with a sound localized in the same region of space, gaze shift occurs. After training, light alone produced a gaze shift (adapted from [41,42]).

The notion that dLGN neurons do not express plasticity was disproved a decade after Wiesel and Hubel’s publication. Indeed, Ikeda and Wright showed in 1976 that the spatial resolution of dLGN neurons activated by the deviating eye in kittens reared with a squint is considerably reduced compared to that of neurons activated by the normal eye [28]. This result was the first to suggest that loss of normal binocular vision leads to plastic changes in the LGN. Later on, it was shown in amblyopic patients that functional deficits in visual response are already observed at the stage of the dLGN [29]. In addition, in contrast to what was previously assumed, about half of the rodent dLGN relay neurons in a given monocular territory in fact receive inputs from both eyes, indicating a potential binocularity for a large proportion of dLGN neurons [30,31,32,33,34,35,36]. Moreover, spatial receptive fields at eye opening in mouse dLGN are ~2 times larger than in adulthood due to an increase in surround suppression owing to an increased in feed-forward inhibition [37]. Furthermore, monocular deprivation (MD) in the mouse has been shown to produce a large shift in ocular dominance (OD) in dLGN neurons (Figure 2A) [38,39,40]. In one of these studies, GABAergic synaptic inhibition was found to be critical [39]. It is very unlikely that the plasticity observed in the dLGN only represents altered feedback from the cortex, because the shift in dLGN responses was resistant to cortical inactivation using the GABA_A_ receptor agonist muscimol [38].

### 2.2. Functional Plasticity in the SC

The SC is the mammalian equivalent of the optic tectum in inferior vertebrates [5]. While many studies reported functional and synaptic plasticity in the tadpole optic tectum [43,44,45,46], fewer investigations have been performed on the mammalian SC. As for the dLGN, the SC was thought to be largely devoid of functional plasticity, since receptive field features were found unchanged after MD [47]. However, recent findings suggest that SC express functional plasticity. The best demonstration of SC plasticity comes from studies on hemianopia, a permanent visual deficit caused by cortical trauma [41,42,48,49]. Patients with unilateral hemianopia are totally blind in the contralateral visual hemi-field but have preserved subcortical visual structures such as the SC. In basic post-traumatic conditions, hemianopia patients display a total lack of gaze orientation towards the blind hemi-field; a behavioral response depending on cortico-collicular connections. However, when the visual stimulus was temporally paired with an auditory stimulus occurring in the same region of the visual space (i.e., audio-visual training), normal gaze orientation towards the blind hemi-field (Figure 2B) was observed both in patients [41] and cats [42,49]. Interestingly, the re-emergence of visual behavior in cats is correlated with the reinstatement of visual responsiveness in deep layer neurons of the ipsilesional SC [42]. This audio-visual training procedure is thought to be related to the Hebbian learning and to reflect potentiation of visually activated synapses onto gaze-orientation related premotor neurons within the SC that fired under the conjoint activation of auditory synapses. In fact, audio-visual training has been shown to be able to reveal auditory or visual responses that were absent initially [50]. 

Working memory is classically thought to result from persistent activity in neuronal circuits or in neurons [51,52]. The entorhinal cortex is thought to be the principal brain locus of working memory [53]. Recent work, however, indicates that the SC is also involved in working memory. In fact, human SC has been shown to participate in a loop of persistent activity possibly supporting working memory [54]. 

Adaptation of the saccade classically involves the cerebellum [55]. However, the SC has also been involved in saccade adaptation as a provider of error signals between the desired and actual movement. Inactivation of the SC by infusion of the GABA_A_ receptor agonist muscimol impairs saccade motor learning in monkeys [56], indicating that intact SC is required for saccade adaptation and that the error signal in this process is provided by the SC.

## 3. Structural Plasticity in Subcortical Visual Areas

### 3.1. Structural Plasticity in the dLGN

**Figure 3:**
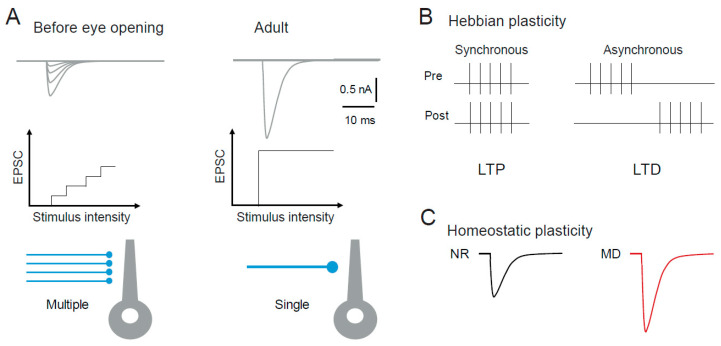
Plasticity in the dLGN. (**A**) Structural plasticity. Refinement, synapse elimination and synapse strengthening at rodent retino-geniculate inputs during postnatal development. Synaptic currents evoked by increasing stimulus intensity before eye opening (left) and in the adult (right). Note the multiple and small synaptic responses in immature dLGN neurons and the all-or-none and large response in mature dLGN neurons. (**B**) Hebbian synaptic plasticity at retino-geniculate synapses induced by pairing presynaptic stimulation with postsynaptic firing with synchronous (left) or asynchronous (right) relation. Adapted from [62]. (**C**) Homeostatic plasticity at cortico-geniculate synapses. MD induces an up-regulation of synaptic transmission. Adapted from [63]. NR, normal rearing.

The visual system is immature at birth and several structural plasticity phenomena occur during early post-natal development, but also at later ages. In particular, a profound reorganization occurs at the retino-geniculate synapse during early post-natal development. For instance, the number of retinal ganglion cells innervating a relay neuron of mouse dLGN decreases from about 10 before eye opening to 1 at the adult stage [57,58] (Figure 3A). This refinement occurs mainly by means of synapse elimination, synapse strengthening and clustering of synaptic boutons [59]. In addition, the dendritic tree in both relay neurons and interneurons evolves during the first weeks of post-natal development, from small to large arborization with a transient peak in dendritic complexity at the time of eye opening [60,61]. 

These maturation processes depend on visual activity. In particular, the dLGN as a whole depends on visual inputs to establish and maintain itself as a functioning structure. The major effect of altering normal vision by suppressing inputs from one eye is to induce the degeneration of downstream visual structures. This has been well documented at the level of the dLGN, where different paradigms of visual deprivation translate into the shrinkage of dLGN relay cells axons, loss of thalamic cells, reduction in soma size and reduction in dLGN volume compared to non-deprived counterparts [23,64,65,66,67,68,69,70]. The effect of visual deprivation on dLGN relay cell dendritic trees has also been characterized. Both in a mouse model of glaucoma and in mice lacking retinal ganglion cells axons, dendritic trees of surviving relay cells ended up showing a strong reduction in size and complexity after an eventual intermediary phase of exuberant branching [70,71,72]. Similarly, albinism or dyslexia reduces the size of the dLGN [73,74]. Altogether, developmental and deprivation studies show that retinogeniculate projections have a trophic and necessary role in establishing and maintaining the morpho-functional complexity of dLGN neurons. 

### 3.2. Structural Plasticity in the SC

Structural plasticity has also been reported in the SC during post-natal development. Retino-collicular synapses in adult rodents follow the retino-topic organization [75]. Early studies have shown that this topographic organization is preserved upon partial lesions of the SC, thanks to an orderly compression of the entire retinal projections onto the remaining SC [76]. Conversely, following partial retinal lesion, remaining axons connect their correct targets to maintain the retinotopic map [77]. These studies suggest that complex signaling mechanisms exist to dynamically establish the topographic organization of retino-collicular connections. However, during early development, retinal axons transiently branch and arborize in inappropriate regions of the SC [78], resulting in topographically diffuse retinal projections. Thus, the ordered projection found in adults ultimately emerges after competitive interactions between retino-collicular contacts. Parallel to this refinement, a transient increase in the complexity of the dendritic tree has been reported in the binocular region of the mouse SC, one week before eye opening [79]. In rodents enucleated at birth, the topographic structure of the retinal projection to the SC is altered, showing that the establishment of an adult topographic map within the SC is mediated by plastic mechanisms during development that depend on visual inputs [80]. 

## 4. Synaptic Plasticity in Subcortical Visual Areas

### 4.1. Synaptic Plasticity in the dLGN

#### 4.1.1. Hebbian Synaptic Plasticity in the dLGN

Hebbian synaptic plasticity at the retino-thalamic synapse was first demonstrated in 2007 by the group of Carla Shatz [62]. Patterned spontaneous activity in the developing retina is known to drive synaptic refinement in the dLGN before eye opening [81,82,83,84]. Using burst-based activity patterns mimicking retinal waves, Shatz and colleagues showed that, before eye opening, synaptic plasticity at retinogeniculate synapse depends on the relative timing between pre- and post-synaptic bursts [62]. Following the Hebbian principle reported at hippocampal synapses [85,86], coincident bursts produced long-term synaptic potentiation (LTP), whereas non-overlapping bursts produced mild synaptic long-term depression (LTD) [62] (Figure 3B). Such LTP induced by pre- and post-synaptic synchronous bursts is likely to be involved in the stabilization of the winner synapses during synaptic refinement. On the other hand, LTD induced by asynchronous burst is likely to reflect preliminary steps leading to synapse elimination [87]. Supporting this idea, deletion of two proteins from the major histocompatibility complex class I (MHC I) required in CNS development and plasticity [88,89,90,91,92] not only suppresses synapse elimination, but also eliminates LTD [93].

#### 4.1.2. Homeostatic Synaptic Plasticity in the dLGN

Homeostatic regulation of synaptic transmission has been reported in dLGN neurons following MD in mice [63]. Interestingly, homeostatic regulation occurs on cortico-thalamic inputs but not on retino-thalamic inputs, suggesting that MD introduces a complex redistribution of synaptic weight in dLGN relay neurons (Figure 3C). In this case, presynaptic release probability was found to be higher at the cortical input on the deprived side [63]. However, no change on the post-synaptic side has been reported. 

### 4.2. Synaptic Plasticity in the SC

#### 4.2.1. Hebbian Synaptic Plasticity in the SC

While many studies reported long-lasting Hebbian synaptic plasticity in the tadpole optic tectum [43,94,95], fewer investigations were performed on long-term synaptic plasticity in the mammalian SC. The first evidence for LTP induction in the SC was provided 30 years ago in the superficial layer of the SC following electrical stimulation at 50 Hz of the optic tract [96]. Since then, several studies have been devoted to identifying the involved cellular mechanisms [97,98]. Interestingly, eye opening itself induces synaptic potentiation in the SC similar to NMDAR-dependent LTP at retino-collicular synapse [99]. Moreover, the cellular mechanisms triggered by eye opening are the prerequisites for the induction of further LTP in the developing rat SC [100]. However, no investigation has been undertaken, so far, to check whether the excitatory synapse linking visual neurons from the upper layer of the SC to premotor neurons from deeper layer express Hebbian potentiation, as suggested by behavioral studies [41,42,48]. 

LTD has been also reported in the SC. Remarkably, LTD is specifically induced at strong but not weak inputs [101], thus suggesting that cooperativity among many activated synapses is required for LTD induction in this structure. 

#### 4.2.2. Homeostatic Synaptic Plasticity in the SC

Homeostatic synaptic plasticity has been reported in the optic tectum of the tadpole following chronic visual deprivation [102] or mechanosensory stimulation [103]. However, no data about homeostatic plasticity have been reported so far in the SC. 

## 5. Intrinsic Plasticity in Subcortical Visual Areas

Beyond synaptic plasticity, changes in intrinsic neuronal excitability represent the other side of functional plasticity that usually goes hand-in-hand with synaptic modifications [104,105] and possibly participate to developmental plasticity and learning [106,107]. Intrinsic plasticity is generally triggered by synaptic activity (induction phase) that induces plasticity of synaptic transmission in parallel. However, the expression of plasticity of intrinsic neuronal excitability depends on the regulation of voltage-gated ion channels (expression phase) such as hyperpolarization-activated cyclic nucleotide-gated (HCN) channels [108,109,110,111], Nav [112], Kv1 [113] and Kv7 [114] channels. 

Whereas intrinsic plasticity has been extensively studied in the visual cortex [115,116,117,118,119] and in the tectum of amphibians [120,121,122], little is known about plasticity of intrinsic excitability in mammalian subcortical areas. In a mouse model of glaucoma in which ganglion cells partly degenerate, intrinsic excitability in dLGN neurons is enhanced and strongly increases spike output [123], suggesting that activity-dependent plasticity of neuronal excitability can be triggered in these neurons. However, the underlying expression mechanisms remain unknown.

## 6. Molecular Correlates of Subcortical Plasticity

While many studies have been devoted to the molecular characterization of subcortical visual areas of mammals through RNA sequencing [124,125,126,127,128], only a handful of studies have examined implications of molecular actors in activity-dependent plasticity in the amphibian tectum [129] and mammalian subcortical visual areas [130,131,132].

### 6.1. Molecular Categorization

Different types of thalamo-cortical neurons with distinct morphologies, connectivity patterns and conveying different aspects of visual information to the cortex have been identified in the dLGN of mice, cats and primates. However, this functional categorization is difficult to translate into molecular terms.

In thalamic nuclei, three major molecular profiles of thalamo-cortical neurons have recently been identified using RNA sequencing [127]. This categorization cannot be explained by cortical projections but reflects a progressive topographical shift across laterally, intermediate and medially localized thalamic nuclei. Notably, ion channels and receptor profiles were organized along the same lines. As a result, differentially projecting thalamic neurons such as thalamo-cortical neurons of the dLGN and ventrobasal sensory neurons, all located in the most lateral part of the thalamus, express a common specific set of genes [127]. Moreover, gene profiling in the mature dLGN showed that transcriptomic differences between principal cell types are subtle relative to the observed differences in morphology and cortical projection targets [128]. In conclusion, these two studies intriguingly reveal a low diversity in dLGN neuron genes.

### 6.2. Molecular Actors in Retinogeniculate Synapse Refinement

The developing retino-geniculate synapse before eye opening constitutes a classical model to study the molecular mechanisms occurring during synaptic refinement and elimination. Microglial activation participates in these mechanisms [133]. Interestingly, immune response proteins occupy a specific place [134] in the process of synapse elimination through microglia. The complement molecule C1q and its downstream component C3 are involved in retino-geniculate refinement, as knocking out C1q prevents the normal segregation of retinal inputs and maintains multiple innervation [135]. Externalization of phosphatidyl-serine, an amphiphilic brain phospholipid, on the neuronal surface of inactive synapses has been recently identified as an “eat me” signal for microglia-mediated pruning in the dLGN [136]. The major histocompatibility complex (MHC) class I molecule H2-D(b) is necessary and sufficient for synapse elimination in the retinogeniculate system [93]. 

Molecular mechanisms responsible for synaptic maintenance also involve secreted proteins [137]. Among them, neuronal pentraxins (NPs) resemble immune system pentraxin which recognize and eliminate pathogens by utilizing the complement pathways and macrophages in the host. Importantly, NP1/NP2 knock-out mice exhibited defects in the segregation of eye-specific retinal ganglion cell (RGC) projections to the dLGN [138]. Interestingly, synthetic protein complexes composed of extracellular scaffolding proteins such as cerebellin-1 and NP1 restore normal function in mouse models of brain disease [139]. However, it is still unknown whether such complexes are able to restore visual function in MD animals.

Leucine-rich repeat trans-membrane 1 protein (LRRTM1) is a key postsynaptic protein involved in synaptogenesis through induction of presynaptic differentiation in contacting axons [140]. The lack of this protein is associated with neuropsychiatric and neurodevelopmental disorders. In dLGN neurons, LRRTM1 also plays the role of a synaptic organizer. By performing next-generation transcriptome analysis of developing mouse visual thalamus, the LRRTM1 expression was found to be highest at eye opening in dLGN neurons [141]. Genetic deletion of LRRTM1 led to a loss of complex retino-geniculate synapses, reduced retinal convergence in visual thalamus and impaired performance in visual tasks requiring processing multiple elements of the visual field [141]. 

Using single-cell RNA sequencing, the gene encoding the intracellular signaling protein kinase C delta (PKCδ) has been identified as the most highly up-regulated gene in the developing dLGN near eye opening (P10-P16) [124]. Interestingly, PKCδ plays a critical role in growth, differentiation and apoptosis [142].

**Figure 4:**
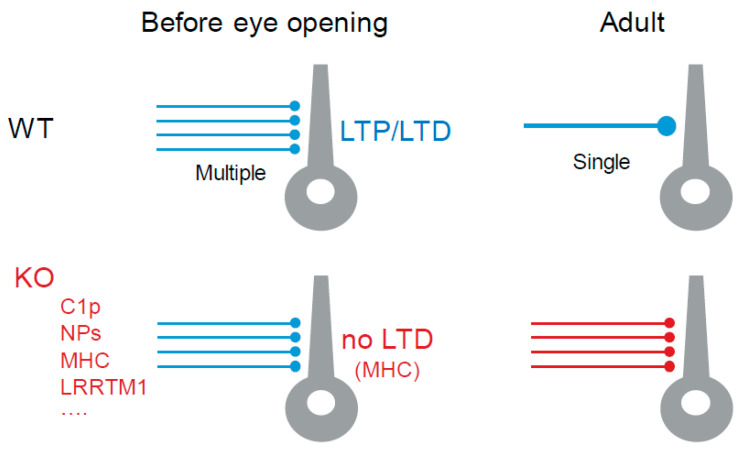
Molecular mechanisms of plasticity in subcortical visual areas. Top, in WT animals before eye opening, thalamocortical neurons are fed by multiple and weak retinal inputs, while in WT adult animals retinal inputs are strong and reduced (refinement). Note that LTP and LTD can be induced at an immature stage. Bottom, in KO animals for proteins involved in synaptic pruning (C1p, NPS, MHC, LRRTM1, and others), no synaptic refinement occurs. Note that LTD is prevented in MHC KO.

In conclusion, deletions of genes coding for C1p, NPs, MHC type 1 and LRRTM1 all prevent synaptic refinement of retinogeniculate inputs (Figure 4). In addition, deletion of one of these genes (MHC type 1) prevents LTD induction [93].

### 6.3. Visual Experience-Dependent Maintenance of Retinogeniculate Connections

Due to a lack of molecular analyses in the late postnatal dLGN, little is known about the mechanisms underlying visual experience-dependent maintenance of retinogeniculate connections [143]. However, a few studies have attempted to fill the gap. Retino-geniculate and cortico-geniculate synapses are provided with different AMPA receptor subunits; while AMPA-type 1 receptor (GluA1) is expressed at retinogeniculate synapses, it is not present at cortico-geniculate synapses [144]. Blocking synaptic activity by means of the local infusion of tetrodotoxin into the dLGN or binocular deprivation reduced GluA1-containing AMPARs trafficking at the retinogeniculate postsynaptic density (PSD) [144]. Another specificity of retino-geniculate inputs is their strong short-term depression that is, in a large portion, mediated by AMPA receptor (AMPAR) desensitization [145]. CKAMP44, an auxiliary subunit of AMPARs, has recently been found to account for most of the short-term synaptic depression observed at retino-geniculate inputs [130]. Furthermore, in mice lacking the transcriptional repressor methyl CpG binding protein 2 (MeCP2), retinogeniculate refinement occurs normally until P21 [146]. However, during a later sensory-dependent phase of synapse development, it results in disruption of connectivity and strength of the retinogeniculate circuit [146]. 

Cytokine receptor fibroblast growth factor-inducible 14 (Fn14), a transcription repressor, is expressed in dLGN neurons. Mice lacking Fn14 showed deficits in synapse refinement: smaller size of retinogeniculate boutons, higher number of PSDs, associated with a higher number of functionally retinogeniculate inputs [125]. In fact, visual experience not only induces Fn14 expression in dLGN relay neurons but also the induction of its ligand TWEAK in microglia [147]. Fn14 increases the number of spines that are not bound to TWEAK-expressing microglia. However, microglial TWEAK bound to Fn14 signals a decrease in synapse number. This microglia-driven loss of synapse occurs through a non-phagocytic mechanism. 

### 6.4. Molecular Analysis of Visual Plasticity

In the optic tectum of the tadpole of *Xenopus laevis*, 83 differentially synthesized proteins that are candidate plasticity proteins have been identified using mass spectrometry [129]. These proteins are involved in a wide range of biological function, including protein translation, RNA splicing and chromatin remodeling. Functional analysis shows that eukaryotic initiation factor three subunit A (eIF3A), the RNA binding protein fused in sarcoma (FUS), and ribosomal protein s17 (RPS17) are required in experience-dependent structural plasticity in tectal neurons. It will be important to verify whether these new candidate plasticity proteins also play an important role in activity-dependent plasticity in the mammalian visual system. 

Neuronal nogo-66 receptor 1 (NgR1) is a high affinity receptor for Nogo, a protein of the myelin that inhibits axon outgrowth. Interestingly, NgR1 is expressed throughout the visual system, including the retina, dLGN, and V1. NgR1 is required to close the critical period of MD-induced plasticity in the cortex [148,149]. Deleting *ngr1* gene in the thalamus is insufficient to restore eye dominance in the cortex following MD but yields an improvement in acuity to normal [131].

## 7. Conclusions

The analysis of the current knowledge on activity-dependent plasticity leads to important conclusions. First, the role of dLGN neurons is not limited to a simple relay linking the retina to the cortex but dLGN neurons are capable of complex integration of visual signals arising from the retina, the cortex and the SC. dLGN neurons also participate to cognitive functions and do express functional and synaptic plasticity. Similarly, SC neurons also display cognitive functions, express functional and synaptic plasticity and have recently been shown to be involved in the recovery from hemianopia, in working memory and in saccade adaptation. 

Many questions remain unsolved. First, the molecular mechanisms underlying functional plasticity are underexplored, and further studies will be required to decipher the key molecular actors involved in functional plasticity in subcortical visual areas. Second, while synaptic plasticity at excitatory synapses have been characterized in subcortical visual areas, little is known about inhibitory synaptic plasticity [150]. Third, the plasticity of intrinsic neuronal excitability has not been explored in detail in the dLGN and the SC. There is no doubt that many of these questions will be addressed in the future.
